# The Impact of Aspirin in Brain Tumor Surgery: To Stop or Not to Stop?

**DOI:** 10.7759/cureus.51231

**Published:** 2023-12-28

**Authors:** Jenny C Kienzler, Javier Fandino

**Affiliations:** 1 Department of Neurosurgery, Kantonsspital Aarau, Aarau, CHE; 2 Department of Neurosurgery, Hirslanden Medical Center Aarau and Zurich, Aarau, CHE

**Keywords:** rebleeding, intracranial hemorrhage, platelet function assay, aspirin, brain tumor surgery

## Abstract

Given the lack of guidelines regarding perioperative management of neurosurgical patients taking antiplatelet medication, a break of aspirin intake for elective brain surgery is recommended. To the best of our knowledge, only three clinical studies have been published comparing re-bleeding rates in patients undergoing elective brain surgery with and without aspirin.

We present a case of an 81-year-old woman who was admitted for elective craniotomy and brain metastases resection. She presented with a right-sided hemianopsia for > two weeks and further investigation by magnetic resonance imaging (MRI) showed the left occipital lesion. For primary cardiovascular prevention, the patient was prescribed prophylactic low-dose aspirin 100 mg. A platelet function test on the day of admission detected highly pathological values. Surgery was scheduled the next day, and aspirin intake was paused. The platelet function test was repeated the morning before surgery. Interestingly, the test showed a 20% above-normal level platelet function. Craniotomy and tumor resection were performed in a routine fashion and no increased bleeding tendency was reported intraoperatively. Postoperatively, the right-sided hemianopsia was immediately regressive. MRI performed 24 hours after surgery demonstrated a complete tumor resection without any signs of rebleeding. The patient was discharged five days after surgery without any neurological deficits.

The literature is limited and guidelines are missing on the topic of management of antiplatelet medication in elective brain surgery. As confirmed by the present case and a review of the literature, elective craniotomy and tumor resection under antiplatelet medication may be considered in certain cases with risk and benefit stratification. More data and randomized controlled trials are needed to confirm these findings.

## Introduction

The management of aspirin and its discontinuation for elective brain tumor surgery is not yet standardized [1-4]. Several authors recommended discontinuation of aspirin for seven days before elective surgery [2], due to the increased risk of intraoperative bleeding or postoperative rebleeding [5]. Nevertheless, other studies have indicated that patients under aspirin had no difference regarding re-bleeding rates [1,3,6]. In elective vascular neurosurgical procedures, such as aneurysm clipping or microvascular decompression, aspirin can be continued directly after surgery without safety concerns. In daily clinical practice, the decision to stop aspirin prior to craniotomy for brain tumor surgery is difficult due to associated comorbidities such as drug-eluted stenting and coronary artery disease [7,8]. We present a case report of a patient taking aspirin for primary cardiovascular prevention due to coronary artery and peripheral arteriosclerotic disease. She was admitted for elective brain tumor surgery and underwent the procedure without any complications. Our case report adds value to the sparse literature and confirms the feasibility of brain tumor resection during aspirin intake. A case illustration and literature review are presented.

## Case presentation

An 81-year-old woman was admitted with the diagnosis of an occipital left tumor. Preoperatively, a metastasis of a known non-small cell lung cancer (NSCLC; adenocarcinoma) was suspected. Video-assisted thoracoscopic lobectomy of the superior right lobe with radical mediastinal lymphadenectomy (pT1A (2 cm) pN0 (0/17) L0 V0 pN0 R0 M0 G2-3) was performed two years prior.

**Figure 1:**
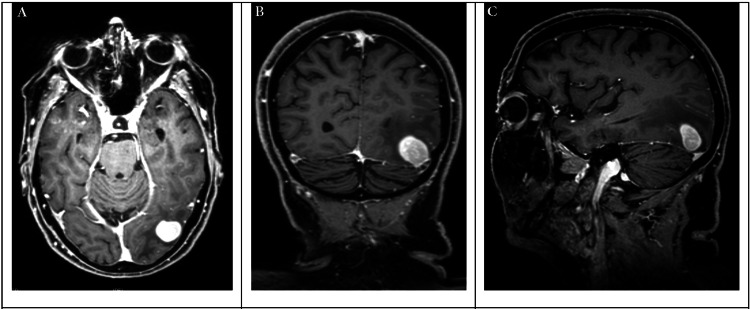
Presurgical imaging Preoperative T1-weighted head MRI images with contrast. (A) Axial, (B) coronal, and (C) sagittal view.

At the time of admission, the patient complained about blurred vision for more than two weeks. Clinical examination revealed a hemianopsia to the right. MRI revealed a 1.7 x 1.1 x 2 cm lesion (Figure 1).

**Figure 2:**
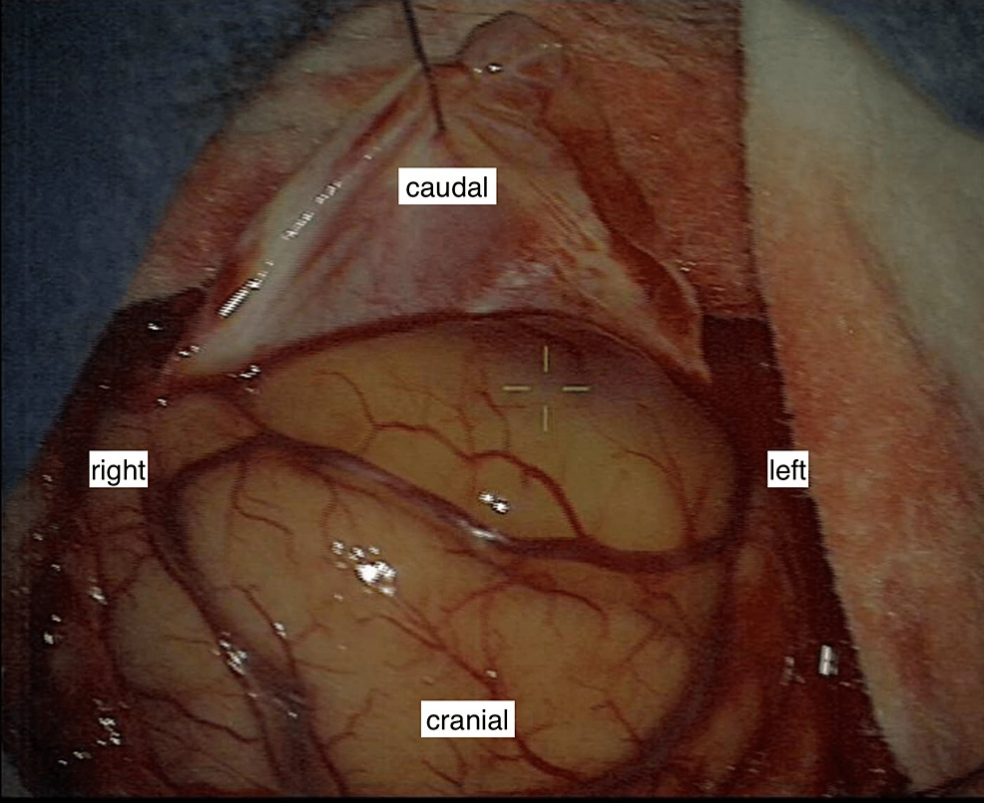
Intraoperative microscopic view Intraoperative picture of the occipital cortex using the Pentero microscope (Carl Zeiss AG, Baden-Württemberg, Germany). The patient was positioned in the Concorde position.

On admission, the patient was receiving aspirin for primary cardiovascular prevention of coronary and peripheral arteriosclerotic disease. We performed a platelet function test, which revealed the following highly pathologic values: collagen/adrenalin ratio was > 300 seconds (reference: 82-150 seconds), and collagen/adenosine diphosphate (ADP) ratio was 93 seconds (reference: 62-100 seconds). Craniotomy and tumor resection were scheduled for the next day, aspirin was discontinued, and the platelet function test was repeated two hours before surgery. Remarkably, both values improved: the collagen/adrenalin ratio decreased to 190 seconds, which is 20% above the normal range, and the collagen/ADP ratio decreased from 93 to 83 seconds. Occipital craniotomy and microscopic resection of the tumor were performed in a standardized manner. Normal hemostasis and bleeding rates associated with resection of the tumor were observed during surgery (Figure 2).

**Figure 3:**
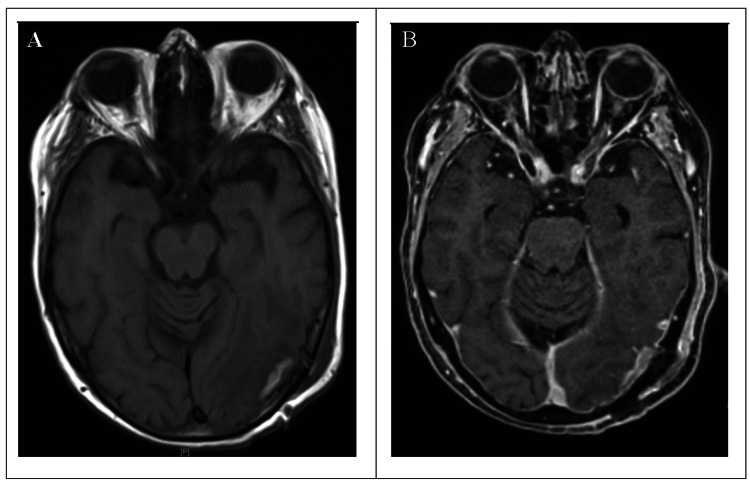
Imaging after surgical resection (A) Axial postoperative T1-weighted native MRI image. A small superficial hyperintense collection of blood is visible, but no relevant postoperative bleeding can be seen. (B) Axial postoperative T1-weighted MRI image with contrast, showing no relevant enhancement and gross total tumor resection.

The documented blood loss was ≤100 milliliter. No postoperative hemorrhage was observed in the MRI the day after surgery (Figure 3).

The patient reported an entire regression of the right-sided hemianopsia. Histological analysis confirmed a metastasis of the NSCLC. The patient was discharged five days after surgery without any neurological deficits. The interdisciplinary tumor board recommended further stereotactic radiation treatment to the resection cavity.

## Discussion

We present a case of successful elective brain tumor surgery, with ultra short-term discontinuation of antiplatelet medication. Aspirin intake in patients scheduled for elective craniotomy and brain surgery is a common phenomenon nowadays.

Elective intracranial surgery

Several groups demonstrated that the risk of increased intraoperative bleeding or postoperative rebleeding is lower with continuous antiplatelet medication, compared to the risk of cerebrovascular thromboembolic events by discontinuation [9]. Di Minno et al. [10] recommended a change of the common practice to stop antiplatelet medication five to 10 days prior to surgical procedures. In their opinion, most surgical procedures can be performed with low-dose aspirin. Only for surgeries with an expected excessive blood loss, aspirin should be stopped on time.

Rahman et al. [1] evaluated the safety of continuing aspirin in their cohort of patients undergoing brain tumor resection from 2010 to 2014. In a total of 452 patients, 368 had no aspirin, 55 discontinued aspirin before surgery, and 28 patients continued aspirin perioperatively. Interestingly, no statistical differences regarding outcomes and complications, such as rebleeding, reoperation, or thromboembolic events, were reported. However, perioperative low-dose aspirin was not associated with an increased risk of complications. These results are in accordance with our findings. A survey on opinions and working practices of 210 neurosurgical facilities in Germany was performed by Korinth et al. [2], investigating the management of patients presenting with low-dose aspirin prior to elective intracranial surgery. Out of 65.7% valid responses, 80.4% of the neurosurgical departments had the policy to preoperatively discontinue aspirin for a mean time of 7.3 days. A similar effect was also observed in a retrospective study by Hanalioglu et al. [6], who analyzed 1291 patients, undergoing 1346 operations. A total of 1068 patients without aspirin and 223 patients with aspirin intake were enrolled. Of these 223 patients, 104 patients paused aspirin at least seven days prior to surgery, and 119 patients continued taking aspirin. Perioperative aspirin continuation was not associated with an increased rate of intraoperative blood loss or hemorrhagic complications following brain tumor surgery [6]. In another study, Kamenova et al. [4] as well as Mahaney et al. [11] compared the peri- and postoperative rebleeding rates associated with aspirin intake in patients undergoing elective ventriculoperitoneal shunt placement. Low-dose aspirin medication intake was present in 23.3%. No statistically significant differences were present between the groups in terms of hemorrhagic events or any other complications.

The issue of discontinuation of aspirin in elective brain surgery is still controversial. We performed a literature review based on publications listed in the US National Library of Medicine National Institutes of Health (PubMed) with the following keywords: “brain tumor surgery,” “intracranial surgery,” “aspirin,” “low-dose aspirin,” “platelet function,” and “hemorrhage.” A total of 19 publications published in the period from 1993 to 2020 were identified (major publications are listed in Table 1). The review of the literature obtained only two publications providing data on the subject of brain tumor surgery and aspirin intake (Table 1) [1,6].

**Table 1:** Review of the literature Overview of publications identified in the literature search associated with aspirin and a neurosurgical intervention.

Author	Year	Region of interest	Procedure	Study type
Hanalioglu et al. [6]	2019	Brain	Elective craniotomy for brain tumors	Interventional
Soleman et al. [12]	2016	Spine	Spinal decompression without instrumentation	Interventional
Greuter et al. [13]	2019	Brain	Emergency craniotomy for traumatic brain injury	Interventional
Kamenova et al. [4]	2016	Brain	Ventriculoperitoneal-shunt placement	Interventional
Rahman et al. [1]	2015	Brain	Elective craniotomy for brain tumors	Interventional
Mahaney et al. [11]	2013	Brain	Ventriculoperitoneal-shunt placement	Interventional
Korinth et al. [14]	2007	Spine	Elective spinal surgery - survey	Observational
Korinth et al. [2]	2006	Brain	Elective intracranial surgery - survey	Observational
James et al. [3]	1997	Brain	Elective intracranial surgery - survey	Observational

Traumatic intracranial hemorrhage

Lee et al. [15] investigated the effect of aspirin in a retrospective study with a cohort of 171 patients ≥ 65 years old. The study cohort underwent emergent surgery for traumatic intracranial hemorrhage (subdural, extradural, or intraparenchymal hemorrhage), and low-dose aspirin medication was neither associated with an increase of perioperative bleeding, length of hospital stay, nor in-hospital mortality. The same results were confirmed in a study by Greuter et al., who analyzed patients under aspirin or anticoagulants undergoing craniotomy or craniectomy because of traumatic brain injury [13]. A surprisingly high number of patients with a Glasgow Coma Scale score of 15 were found to have a traumatic intracranial hemorrhage (11.5% of patients in the aspirin-treated group and 16.5% of patients in the control group) in a study by Spektor et al. [16], who investigated the effect of aspirin in traumatic brain injuries. However, surgery was not necessary for any of these patients. There was no statistically significant difference in frequency or type of traumatic intracranial hemorrhage between patients who were under aspirin use and those without. The authors concluded that low-dose aspirin does not increase parenchymal or subdural bleeding to a surgical relevant point following moderate and minor head injury in patients > 60 years old.

Investigating the recurrence of chronic subdural hematoma, Kamenova et al. [17] were able to exclude any significant association with early postoperative resumption of low-dose aspirin. In addition, cardiovascular events, surgical morbidity, and mortality did not significantly differ between patients with or without aspirin.

The results of these studies support the fact that continuation of aspirin treatment prior to elective and emergent brain surgery is not associated with a higher rate of bleeding. It is noteworthy that 7% of patients treated with aspirin and 20% of patients treated with clopidogrel who are admitted with a traumatic intracranial hemorrhage are so-called "non-responders" to antiplatelet drugs [18]. In this context, it should also be mentioned that certain generic aspirin drugs may have different pharmacokinetics and effects. However, an error in a platelet function test in the laboratory could also lead to misleading results. Metastatic cancer per se could also have an influence on platelet function and this needs further investigation.

Platelet concentrate transfusion

To investigate the rescue mechanisms, Li et al. [19] conducted a prospective, randomized, controlled trial in patients with basal ganglia hypertensive hemorrhage undergoing craniotomy and hematoma removal with or without aspirin to examine the effects of aspirin use and concomitant transfusion of apheresis concentrate on postoperative bleeding and mortality rates. The transfusion of platelets reduced the postoperative hemorrhage rate, the average hemorrhage volume, and disability and mortality rate in aspirin responders. On the contrary, Downey et al. [20] analyzed a population of 328 patients with traumatic brain injury and documented the use of clopidogrel or aspirin prior to injury. Patients with (n = 166) and without (n = 162) platelet transfusions were compared in terms of mortality rates. Patients with platelet transfusion revealed a mortality rate of 17.5%, compared to 16.7% without, and therefore transfusion of platelets in this study did not reduce mortality.

## Conclusions

Management of aspirin in brain tumor surgery is still lacking specific guidelines. Based on this review of the literature and case illustration, aspirin continuation before elective brain surgery may be considered in certain cases with risk and benefit assessment. Especially in high-risk patients, antiplatelet therapy cannot be discontinued to prevent cardiovascular complications. However, prospective randomized trials are needed to provide more evidence for the safety of aspirin use in brain tumor surgery.
